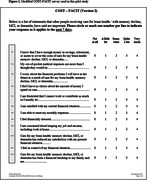# Assessment of Financial Toxicity in an ADRC Cohort: a Pilot Study

**DOI:** 10.1002/alz.085230

**Published:** 2025-01-09

**Authors:** Radmila Choate, Justin M Barber, Gregory A Jicha, Erin L. Abner

**Affiliations:** ^1^ University of Kentucky Sanders‐Brown Center on Aging, Lexington, KY USA; ^2^ University of Kentucky College of Public Health, Lexington, KY USA; ^3^ Sanders‐Brown Center on Aging, Lexington, KY USA; ^4^ University of Kentucky College of Medicine, Lexington, KY USA; ^5^ College of Public Health, University of Kentucky, Lexington, KY USA; ^6^ Sanders‐Brown Center on Aging, University of Kentucky, Lexington, KY USA

## Abstract

**Background:**

Alzheimer’s disease and related dementias (ADRD) are associated with substantial direct healthcare costs, including specialist care, medication, and indirect costs related to loss of productivity and informal caregiving. The economic burden of ADRD on families and caregivers often threatens the financial security of entire households. The consequences of this financial burden are linked to a worsened quality of life and treatment compliance, rapid disease progression, and lower survival. The term “financial toxicity” is widely used in cancer research to describe the impact of financial hardship due to cancer treatment. However, financial toxicity remains understudied in people living with ADRD, their families, and caregivers.

**Methods:**

We used the 12‐item **Co**mprehensive **S**core for Financial **T**oxicity v.2 (COST‐FACIT) instrument to measure financial distress related to receiving care. 11 of the 12 items are used to create a financial toxicity score (range 0‐44), with higher scores indicating better financial well‐being (Figure 1). The instrument used in this study was adapted for use in individuals with normal cognition and cognitive impairment. The data were collected electronically from the University of Kentucky Alzheimer’s Disease Research Center cohort, a convenience sample of community‐based older adults across the spectrum of cognition followed longitudinally until death. We used descriptive statistics to characterize the survey respondents and evaluate their self‐perceived financial distress.

**Results:**

These preliminary analyses include data from 153 adults who responded to the emailed survey. The mean age of the cohort was 79.4 years (SD = 6.3), 70% female, 93% white, with an average of 17 years of education; 138 (90%) had normal cognition, and 15 (10%) had MCI. The average COST‐FACIT score (range 0‐44) was 32.6 (SD = 7.7) in the overall cohort, indicating a low grade of financial toxicity. We did not observe an association between self‐perceived financial burden and age, sex, years of education, or cognitive status in this cohort of older adults.

**Conclusions:**

Further and larger studies are needed to evaluate financial toxicity across the cognition spectrum and to assess factors associated with high financial burden, including cost‐coping behaviors and health‐related quality of life of people living with ADRD and their families.